# Myocardial deformation imaging by 2D speckle tracking echocardiography for assessment of diastolic dysfunction in murine cardiopathology

**DOI:** 10.1038/s41598-023-39499-3

**Published:** 2023-07-31

**Authors:** L. J. Daniels, C. Macindoe, P. Koutsifeli, M. Annandale, S. L. James, L. E. Watson, S. Coffey, A. J. A. Raaijmakers, K. L. Weeks, J. R. Bell, J. V. Janssens, C. L. Curl, L. M. D. Delbridge, Kimberley M. Mellor

**Affiliations:** 1 Cellular and Molecular Cardiology Laboratory, Department of Physiology, University of Auckland, Auckland, New Zealand; 2 Department of Anatomy and Physiology, University of Melbourne, Melbourne, Australia; 3 Auckland Bioengineering Institute, University of Auckland, Auckland, New Zealand; 4 Radcliffe Department of Medicine, OCDEM, University of Oxford, Oxford, UK; 5 Baker Department of Cardiometabolic Health, University of Melbourne, Melbourne, Australia; 6 Department of Microbiology, Anatomy, Physiology and Pharmacology, La Trobe University, Melbourne, Australia; 7 Department of Medicine, University of Otago, Dunedin, New Zealand

**Keywords:** Cardiovascular biology, Experimental models of disease, Preclinical research, Diabetes, Metabolic syndrome, Obesity

## Abstract

Diastolic dysfunction is increasingly identified as a key, early onset subclinical condition characterizing cardiopathologies of rising prevalence, including diabetic heart disease and heart failure with preserved ejection fraction (HFpEF). Diastolic dysfunction characterization has important prognostic value in management of disease outcomes. Validated tools for in vivo monitoring of diastolic function in rodent models of diabetes are required for progress in pre-clinical cardiology studies. 2D speckle tracking echocardiography has emerged as a powerful tool for evaluating cardiac wall deformation throughout the cardiac cycle. The aim of this study was to examine the applicability of 2D speckle tracking echocardiography for comprehensive global and regional assessment of diastolic function in a pre-clinical murine model of cardio-metabolic disease. Type 2 diabetes (T2D) was induced in C57Bl/6 male mice using a high fat high sugar dietary intervention for 20 weeks. Significant impairment in left ventricle peak diastolic strain rate was evident in longitudinal, radial and circumferential planes in T2D mice. Peak diastolic velocity was similarly impaired in the longitudinal and radial planes. Regional analysis of longitudinal peak diastolic strain rate revealed that the anterior free left ventricular wall is particularly susceptible to T2D-induced diastolic dysfunction. These findings provide a significant advance on characterization of diastolic dysfunction in a pre-clinical mouse model of cardiopathology and offer a comprehensive suite of benchmark values for future pre-clinical cardiology studies.

## Introduction

Diastolic dysfunction is characterized by impaired heart relaxation due to increased myocardial wall stiffness. Identification of early diastolic dysfunction has important prognostic value for progression to heart failure. Heart failure with preserved ejection fraction (HFpEF), a condition characterized by diastolic dysfunction, is highly prevalent in diabetic patients and has worse prognosis than in non-diabetic patients^1,2^. Currently, while there are new advances in treatments for patients with HFpEF, the benefits are largely seen in those with advanced disease^3,4^, thus a substantial research effort is underway for drug-discovery in pre-clinical models. There is an urgent need for validated tools for systematic evaluation of diastolic function in small rodent models of disease. Clinically important conventional echocardiography approaches to assess diastolic function clinically (e.g. flow and tissue Doppler imaging, E/e’) have been applied to tracking diastolic function in small rodents^5,6^, but high heart rates (> 400 bpm) and small cardiac anatomy present unique challenges^7^. A high level of training and expertise is required to generate reliable outcomes. Speckle tracking echocardiography is a post-processing tool to track deformation of the myocardial wall throughout the cardiac cycle, and is increasingly used in both clinical^8–11^ and pre-clinical studies^12–15^. Global cardiac strain is the most commonly reported variable from speckle tracking studies, which is typically associated with characteristics of systolic function. Notably, speckle tracking-derived strain rate can be determined over the full length of the cardiac cycle and peak strain rate during the diastolic period may provide a robust additional measure to support the evaluation of diastolic function in small rodent models for pre-clinical studies^7^.

Speckle tracking echocardiography is based on tracking the motion of speckle patterns created by interference of ultrasound beams in the myocardium over time^16^. The extent and speed of deformation of a specific area of the myocardium is recorded, in relation to the initial dimensions. A significant advantage of speckle tracking echocardiography is that cardiac function can be assessed in long and short axis viewing planes to obtain information on longitudinal, radial and circumferential strain for any given area of interest. Tracking regional changes in myocardial wall deformation has proven to be useful particularly in identifying ischemic regions in studies of myocardial infarction^14,15^. This approach may also be applicable to generating a more advanced understanding of regional cardiac dysfunction in chronic disease states.

Whilst speckle tracking echocardiography is becoming more widely used, particularly for reporting global strain measures, its utility in expanding the available tools for assessment of diastolic function in small rodent models has not been fully realized. Given the escalating prevalence of diabetes, and the predisposition for diastolic dysfunction (and HFpEF) in diabetic patients, diastolic strain rate may be an important measure for pre-clinical studies investigating novel drug targets for treatment of diabetic heart disease. The few studies available reporting diastolic strain rate in diabetic rodent models have mostly used genetic models of advanced diabetes^17–19^. Regional differences in diastolic function have not been examined. An evaluation of speckle tracking echocardiography to monitor diastolic function in more clinically relevant models of diabetes is lacking. Thus the goals of this study were to validate speckle tracking echocardiography as a tool to assess diastolic myocardial wall deformation and evaluate regional differences in diastolic dysfunction in a dietary (high fat high sugar) mouse model of obesity and type 2 diabetes (T2D).

## Results

### Doppler echocardiography confirms diastolic dysfunction in T2D mice

**Figure 1:**
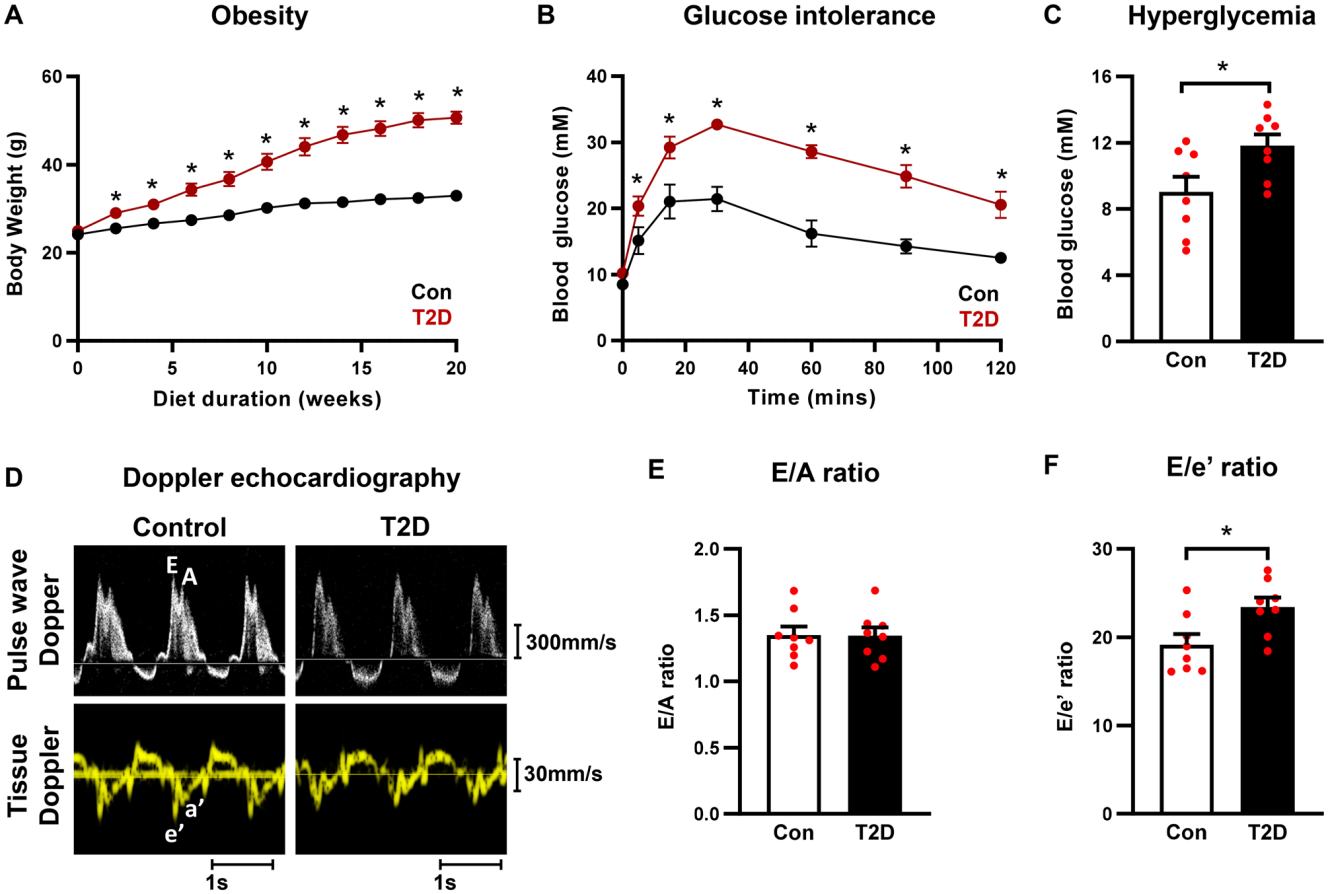
High fat high sugar diet-induced T2D mice exhibit obesity, impaired glucose tolerance, hyperglycemia and diastolic function. (**A**) Body weight progression throughout the 20 week dietary intervention. Note, in some instances error bars are not discernible as they fall within symbol shapes. Analyzed by 1-way repeated measures ANOVA, annotated with LSD post hoc analyses. (**B**) Glucose tolerance test in T2D mice (11 weeks high fat high sugar diet). Note, in some instances error bars are not discernible as they fall within symbol shapes. Analyzed by 1-way repeated measures ANOVA, annotated with LSD post hoc analyses. (**C**) Blood glucose levels in T2D mice at study endpoint (20 weeks high fat high sugar diet). Analyzed by Students unpaired t-test. (**D**) Representative Doppler echocardiography traces for control and T2D mice. Upper panels, mitral valve pulse wave (blood flow) Doppler imaging. Lower panels, mitral annulus tissue Doppler imaging. (**E**) Ratio of pulse wave Doppler E wave to A wave amplitude in T2D mice (20 weeks high fat high sugar diet). Analyzed by Students unpaired t-test. (**F**) Ratio of mitral valve flow Doppler E wave to mitral annulus tissue Doppler e’ wave in T2D mice (20 weeks high fat high sugar diet). Analyzed by Students unpaired t-test. Data are presented as mean ± SEM. n = 8/group. *p < 0.05.

To develop a mouse model of T2D, mice were fed a high fat high sugar diet for 20 weeks. T2D mice exhibited significantly increased bodyweight from 2 weeks of dietary intervention (Fig. 1A), impaired glucose tolerance (11 weeks diet duration, Fig. 1B), and hyperglycemia at study endpoint (20 weeks diet duration, Fig. 1C). Representative pulse wave and tissue Doppler images from control and T2D mice are presented in Fig. 1D. T2D mice showed no change in the ratio of early (E) to late (A) mitral valve blood flow rate (E/A ratio) compared to control mice (Fig. 1E). A modest significant decrease in isovolumetric relaxation time (IVRT) and increase in mitral valve E-wave deceleration time was observed in T2D mice (Supplementary Fig. S3A,B). The commonly used clinical index of ventricular filling (ratio of early (E) mitral valve flow rate to early mitral annulus tissue movement (e’), E/e’) was significantly increased (Fig. 1F), confirming the presence of diastolic dysfunction in this dietary T2D mouse model. The reciprocal and modest (not individually significant) shifts in parameters E and e’ combined in the composite parameter E/e’ to yield a significant effect. Relatively, there was a slightly greater contribution of the reduction in e’ compared to the elevation in E, which underlies the overall change in E/e’. A more detailed timecourse study of these separate parameter shifts in various diabetic disease models could provide further mechanistic insight.

### T2D mice exhibit preserved ejection fraction and reduced global longitudinal strain

**Figure 2:**
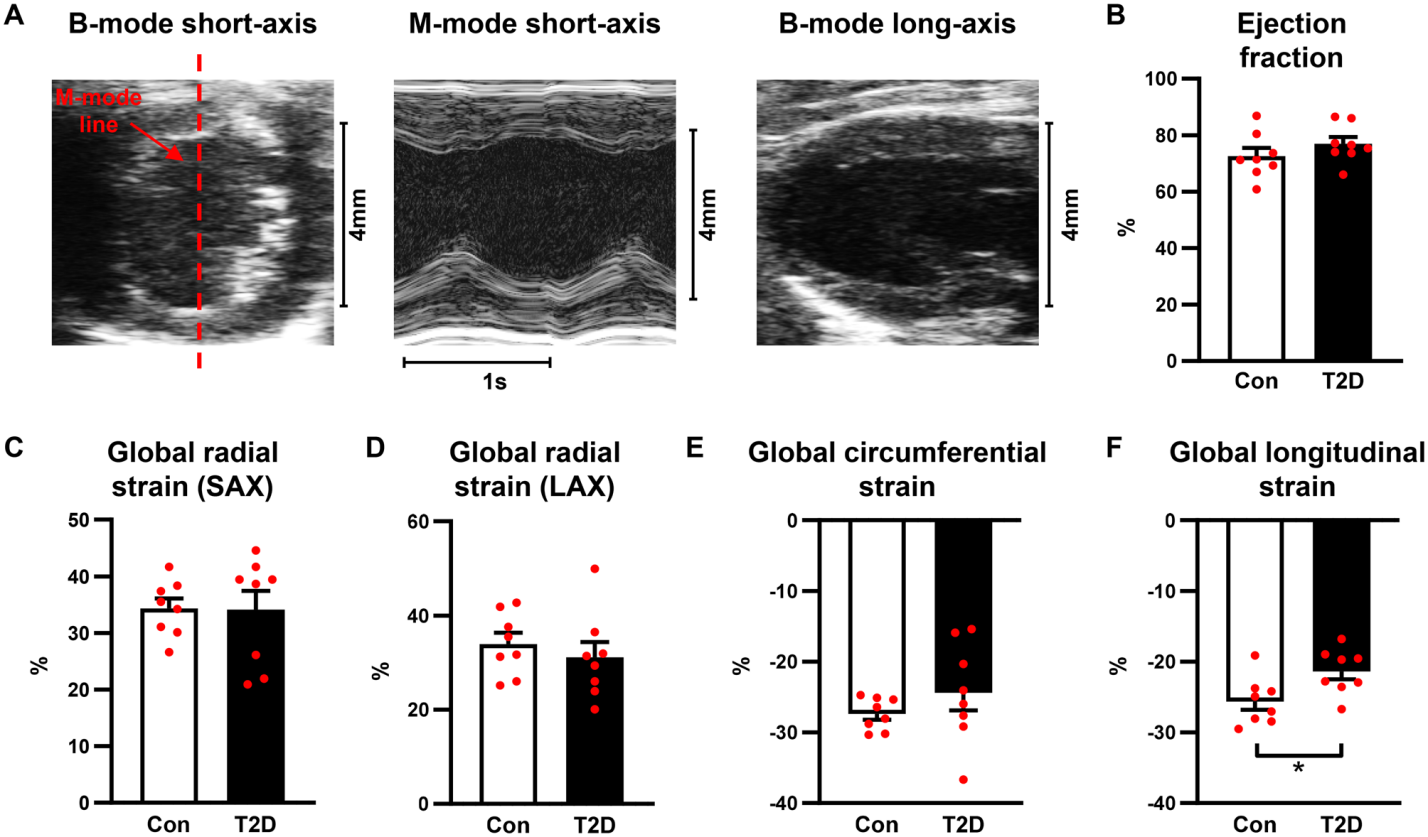
T2D mice exhibit preserved ejection fraction and reduced global longitudinal strain. (**A**) Exemplar M-mode and B-mode traces from the left ventricular short- and long-axis imaging planes. Red dashed line indicates positioning of M-mode line scan. (**B**) Ejection fraction. (**C**) Global radial strain, analyzed in the short axis (SAX) imaging plane. (**D**) Global radial strain, analyzed in the long axis (LAX) imaging plane. (**E**) Global circumferential strain, analyzed in the short-axis imaging plane. (**F**) Global longitudinal strain, analyzed in the long-axis imaging plane. Data are presented as mean ± SEM. n = 7–8/group. Analyzed by Students unpaired t-test, *p < 0.05.

To evaluate cardiac dimensions and systolic function, we performed M-mode echocardiography and B-mode 2D speckle tracking strain imaging in control and T2D mice. Exemplar M-mode and B-mode echocardiography images are shown in Fig. 2A. Left ventricular hypertrophy was detected in T2D mice, as evidenced by increased left ventricular mass, and increased systolic and diastolic posterior wall thickness (Table 1). Systolic function measured via ejection fraction (Fig. 2B) and fractional shortening (Table 1) was not different between control and T2D mice. Global radial strain (measured in the short-axis and long-axis views) and global circumferential strain were not different between control and T2D mice (Fig. 2C–E, Supp Table S1). In contrast, global longitudinal strain measured in the long-axis view was significantly reduced in the T2D mice (T2D: − 21.4 ± 1.1% vs Control: − 25.6 ± 1.2%, p < 0.05, Fig. 2F, Supp Table S1). These data suggest that T2D mice exhibit some evidence of mild longitudinal systolic dysfunction, despite preserved ejection fraction.

**Table 1:** In vivo echocardiography cardiac functional and structural characteristics in T2D mice.

	Control	T2D
Heart rate (bpm)	508 ± 8.5	486 ± 6.7
LV mass (corrected) (mg)	81.6 ± 25	103.6 ± 15*
ESV (μl)	16.3 ± 3.2	12.0 ± 1.3
EDV (μl)	54.1 ± 6.6	50.2 ± 1.9
Ejection fraction (%)	72.6 ± 2.5	76.9 ± 1.9
Fractional shortening (%)	41.8 ± 2.5	45.4 ± 2.3
Stroke volume (μl)	37.8 ± 3.6	38.2 ± 1.1
Cardiac output (ml/min)	19.3 ± 2.0	18.5 ± 0.6
LVAWs (mm)	1.44 ± 0.059	1.66 ± 0.087
LVAWd (mm)	0.876 ± 0.040	1.04 ± 0.049
LVPWs (mm)	1.20 ± 0.046	1.42 ± 0.050*
LVPWd (mm)	0.712 ± 0.022	0.974 ± 0.038*
E wave (mm/s)	628 ± 28	705 ± 29
A wave (mm/s)	477 ± 39	537 ± 37
e’ wave (mm/s)	33.5 ± 2.2	30.4 ± 1.6
a’ wave (mm/s)	24.1 ± 2.1	20.7 ± 1.5

Data are presented as mean ± SEM. n = 8/group.

*ESV* left ventricular end systolic volume, *EDV* left ventricular end diastolic volume, *LVAWs* left ventricular anterior wall thickness at end systole, *LVAWd* left ventricular anterior wall thickness at end diastole, *LVPWs* left ventricular posterior wall thickness at end systole, *LVPWd* left ventricular posterior wall thickness at end diastole, *E wave* mitral valve blood flow velocity during the early ventricular filling phase, *A wave* mitral valve blood flow velocity during atrial contraction, *e’ wave* mitral annulus tissue movement velocity during the early ventricular filling phase, *a’ wave* mitral annulus tissue movement velocity during atrial contraction.

Analyzed by Students unpaired t-test, *p < 0.05.

### Peak diastolic strain rate and peak diastolic velocity are impaired in T2D mice

**Figure 3:**
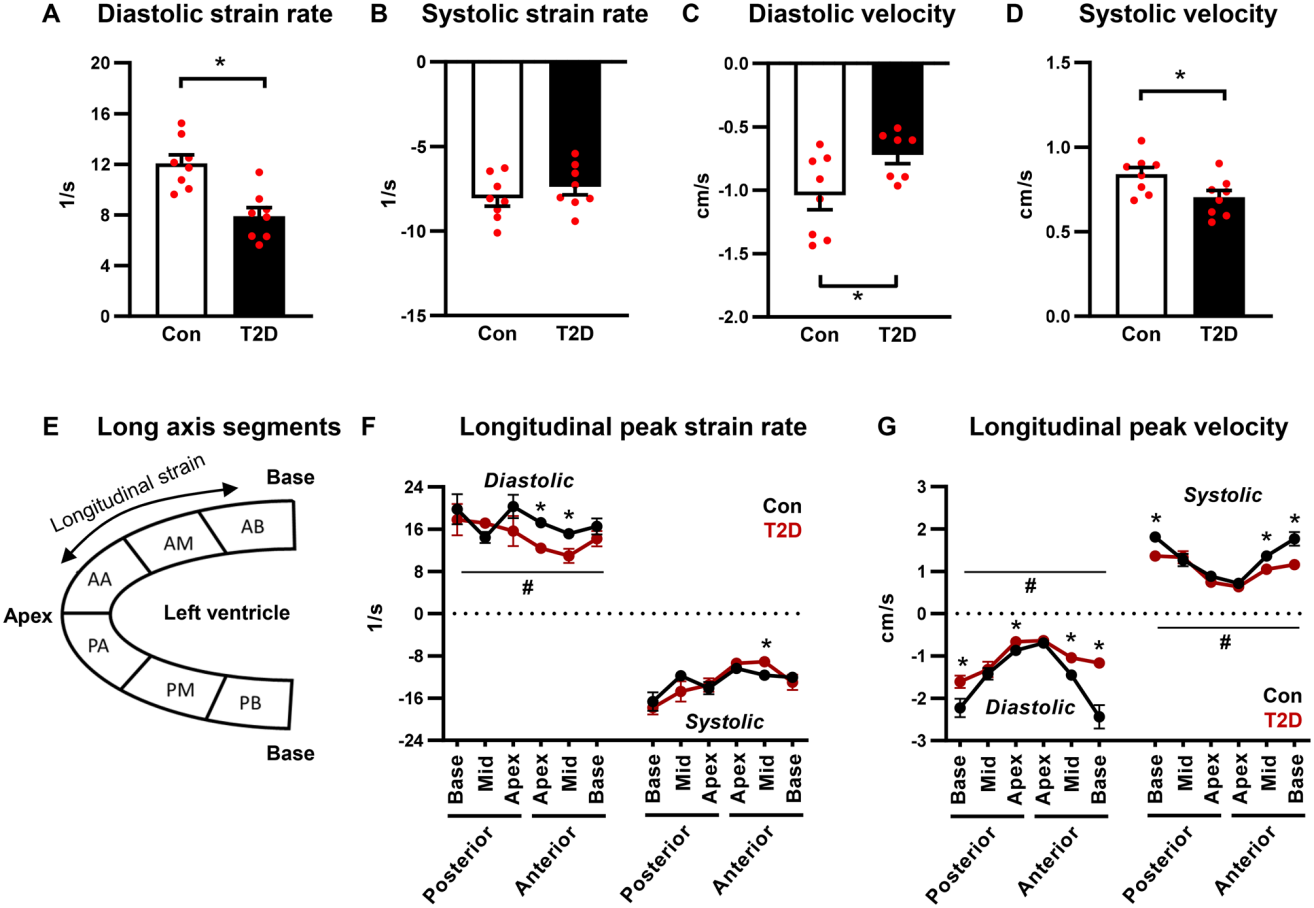
Longitudinal peak diastolic strain rate and velocity are reduced in T2D mice. (**A**,**B**) Longitudinal peak diastolic and systolic strain rate, acquired in the long axis imaging plane. Analyzed by Students unpaired t-test. (**C**,**D**) Longitudinal peak diastolic and systolic velocity, acquired in the long axis imaging plane. Analyzed by Students unpaired t-test. (**E**) Schematic of long axis view left ventricle segmentation for regional strain analysis. (**F**) Regional longitudinal peak strain rate. Analyzed by 1-way repeated measures ANOVA, annotated with LSD post hoc analyses. (**G**) Regional longitudinal peak velocity. Analyzed by 1-way repeated measures ANOVA, annotated with LSD post hoc analyses. Data are presented as mean ± SEM. n = 7–8/group. *p < 0.05 post hoc analysis; ^#^p < 0.05 T2D factor effect. *PB* posterior base, *PM* posterior mid-region, *PA* posterior apex, *AA* anterior apex, *AM* anterior mid-region, *AB* anterior base.

A key advantage of speckle tracking echocardiography is the ability to measure longitudinal, radial and circumferential myocardial deformation throughout the cardiac cycle, and therefore distinct measures of systolic and diastolic ventricular kinetics can be acquired (see representative traces, Supplementary Fig. S1). Longitudinal early peak diastolic strain rate and velocity were reduced in T2D mice (33% and 32% decrease respectively, p < 0.05, Fig. 3A,C, Supp Table S1). Longitudinal peak systolic strain rate was unchanged (Fig. 3B, Supp Table S1) and a small but significant decrease in peak systolic velocity was detected (16% decrease, p < 0.05, Fig. 3D, Supp Table S1). A schematic of regional segmentation of the ventricle in long axis view is shown in Fig. 3E. Regional analysis revealed that changes in longitudinal peak diastolic strain rate were most prominent in the anterior free wall (apex and mid regions, Fig. 3F). Longitudinal peak diastolic velocity was significantly reduced in the posterior base and apex, and anterior mid- and base regions (Fig. 3G). Reduced peak systolic velocity was detected in similar regions (Fig. 3G). An overall significant T2D ANOVA factor effect was evident for reduced longitudinal peak diastolic strain rate, peak diastolic velocity and peak systolic velocity (Fig. 3F–G), consistent with global measures presented in Fig. 3C,D.

**Figure 4:**
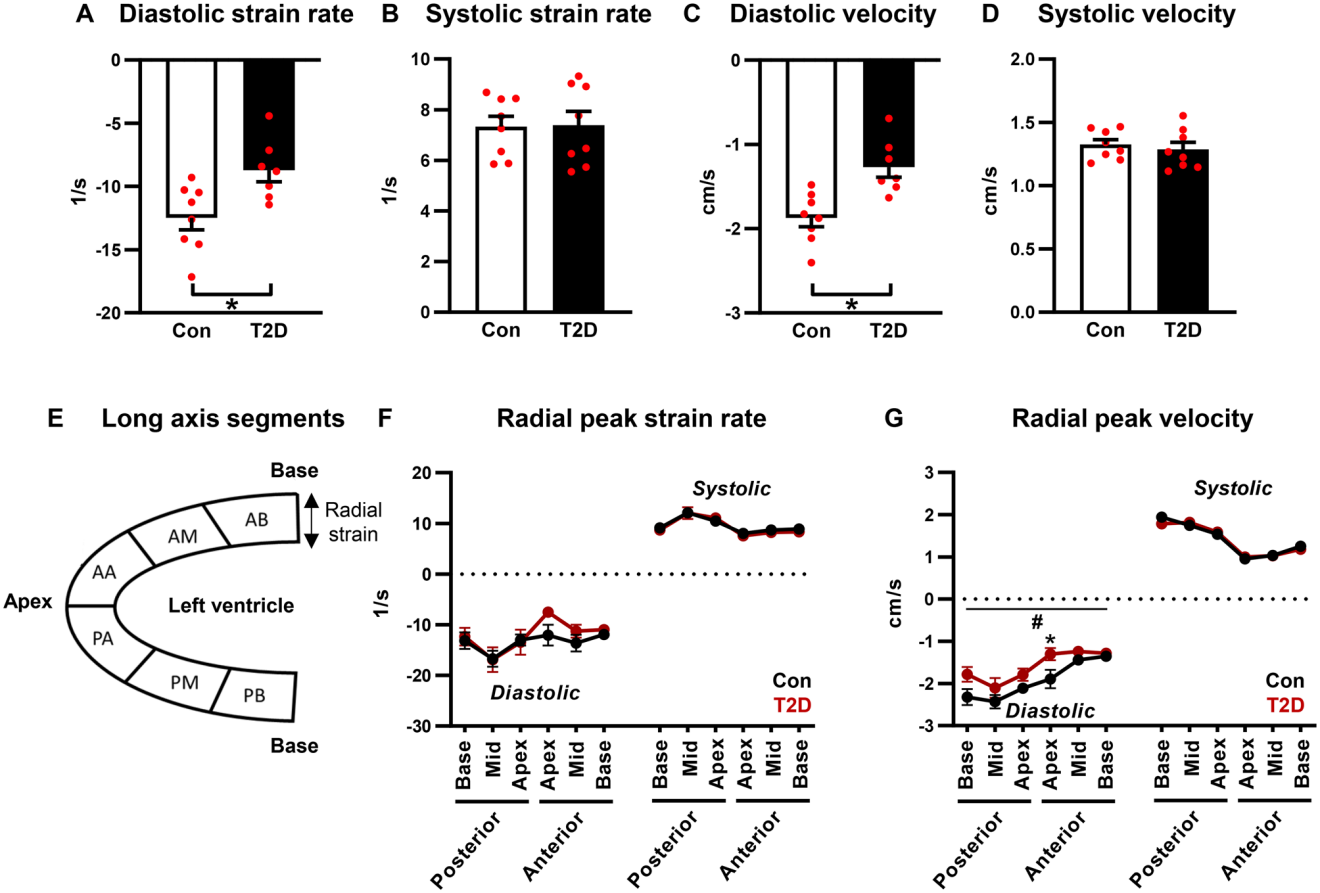
Radial diastolic peak strain rate and velocity are reduced in T2D mice. (**A**,**B**) Radial diastolic and systolic peak strain rate, acquired in the long axis imaging plane. Analyzed by Students unpaired t-test. (**C**,**D**) Radial diastolic and systolic peak velocity, acquired in the long axis imaging plane. Analyzed by Students unpaired t-test. (**E**) Schematic of long axis view left ventricle segmentation for regional strain analysis. (**F**) Regional radial peak strain rate. Analyzed by 1-way repeated measures ANOVA, annotated with LSD post hoc analyses. (**G**) Regional radial peak velocity. Analyzed by 1-way repeated measures ANOVA, annotated with LSD post hoc analyses. Data are presented as mean ± SEM. n = 7–8/group. *p < 0.05 post hoc analysis; ^#^p < 0.05 T2D factor effect. *PB* posterior base, *PM* posterior mid-region, *PA* posterior apex, *AA* anterior apex, *AM* anterior mid-region, *AB* anterior base.

Radial early peak diastolic strain rate and velocity, measured from long-axis images, were reduced in T2D mice (33% and 26% decrease respectively, p < 0.05, Fig. 4A,C, Supp Table S1). Radial peak systolic strain rate and velocity were unchanged (Fig. 4B,D, Supp Table S1). A schematic of regional segmentation of the ventricle in long axis view is shown in Fig. 4E. Regional analysis of radial peak diastolic and systolic strain rate detected no significant differences between control and T2D mice (Fig. 4F). An overall significant T2D ANOVA factor effect was evident for peak diastolic velocity, and this effect was most marked in the anterior apex region (Fig. 4G).

**Figure 5:**
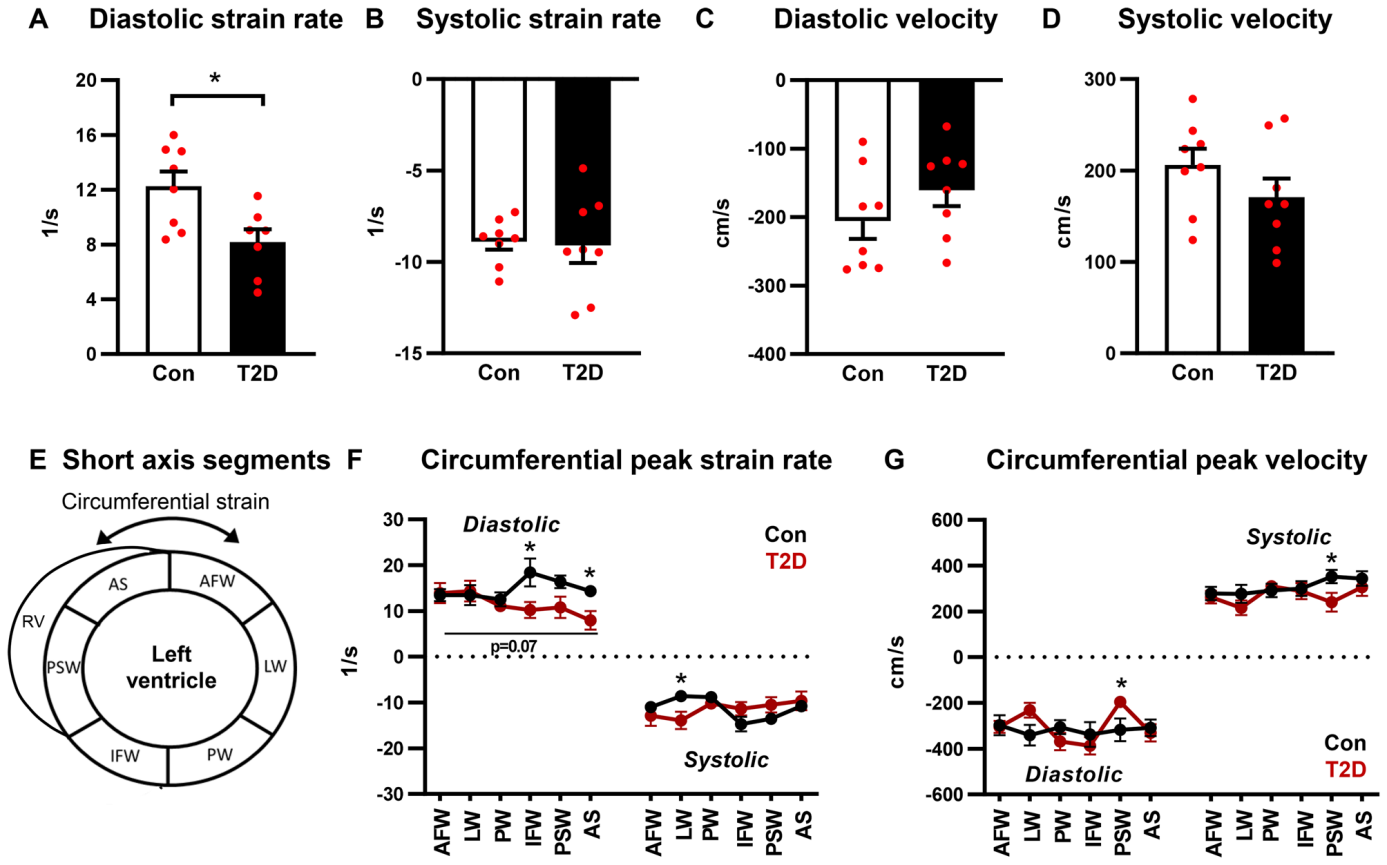
Circumferential diastolic strain rate is reduced in T2D mice. (**A**,**B**) Circumferential diastolic and systolic peak strain rate, acquired in the short axis imaging plane. Analyzed by Students unpaired t-test. (**C**,**D**) Circumferential diastolic and systolic peak velocity, acquired in the short axis imaging plane. Analyzed by Students unpaired t-test. (**E**) Schematic of short axis view left ventricle segmentation for regional strain analysis. (**F**) Regional circumferential peak strain rate. Analyzed by 1-way repeated measures ANOVA, annotated with LSD post hoc analyses. P = 0.07 T2D factor effect. (**G**) Regional circumferential peak velocity. Analyzed by 1-way repeated measures ANOVA, annotated with LSD post hoc analyses. Data are presented as mean ± SEM. n = 7–8/group. *p < 0.05. *RV* right ventricle, *AFW* anterior free wall, *LW* lateral wall, *PW* posterior wall, *IFW* inferior free wall, *PSW* posterior septal wall, *AS* anterior septum.

Circumferential early peak diastolic strain rate was reduced in T2D mice (34% decrease, p < 0.05, Fig. 5A, Supp Table S1) and early peak diastolic velocity was not different between groups (Fig. 5C, Supp Table S1). Circumferential peak systolic strain rate and velocity were unchanged (Fig. 5B,D, Supp Table S1). A schematic of regional segmentation of the ventricle in short axis view is shown in Fig. 5E. Regional analysis of circumferential diastolic peak strain rate detected significantly lower values in the inferior free wall and anterior septum regions (Fig. 5F) and diastolic peak velocity was significantly lower in the posterior septal wall (Fig. 5G). A small but significant decrease in circumferential systolic peak velocity was evident in the posterior septal wall region (Fig. 5G). Collectively these data on myocardial wall deformation kinetics confirm that diastolic dysfunction is detected using peak strain rate and velocity analyses.

### Inter-observer variability

To evaluate the reproducibility of assessing diastolic function via Doppler vs speckle tracking methods, the inter-observer variability of E/e’ ratio and longitudinal early peak diastolic strain rate was analyzed. Both E/e’ ratio and longitudinal diastolic strain rate showed significant correlations between observers (Supplementary Fig. S2A,B). The Pearson correlation coefficient (r) was higher for E/e’ than diastolic strain rate (0.95 vs 0.81) indicative of lower inter-observer variability. Careful monitoring of regional strain data reproducibility in murine settings is important—even clinical speckle tracking reproducibility can be problematic.

## Discussion

This study provides a comprehensive report of global and regional diastolic dysfunction in murine cardiopathology using 2D speckle tracking echocardiography. Peak diastolic strain rate was lower in T2D mice, evident in the longitudinal, radial and circumferential planes. Peak diastolic velocity was lower in T2D mice in the longitudinal and radial planes, but this effect did not reach significance in the circumferential plane. Consistent with the finding that ejection fraction was preserved in this setting, systolic strain rate was unchanged with T2D in all imaging planes. Interestingly, global longitudinal strain, typically considered a measure of systolic function, was significantly impaired in T2D mice, which may provide an indicator of early systolic impairment in the longitudinal plane. Regional analysis of longitudinal strain rate revealed that the anterior free wall of the left ventricle is particularly vulnerable to T2D-induced diastolic dysfunction. These findings deliver important benchmark values for future pre-clinical cardiology studies in the field of diastolic dysfunction, HFpEF and diabetic heart disease.

Although speckle tracking echocardiography was first introduced as a tool for in vivo assessment of myocardial strain in mice over 15 years ago^12,20^, very few studies have used this technique to report specific measures of diastolic function in rodents, i.e. diastolic peak strain rate and diastolic peak velocity. In the present study, decreased longitudinal diastolic peak strain rate and velocity were coincident with decreased E/e’, the commonly reported index of diastolic dysfunction derived from Doppler imaging. Overall, the extent of reduction in diastolic strain rate (longitudinal, radial & circumferential, 33–34% decrease) was larger than the extent of increase in E/e’ detected in diabetic mice (22% increase). The use of high frequency echocardiography for measurement of diastolic strain rate and velocity provides a less angle-dependent method of assessing diastolic dysfunction in settings of fast heart rates in mice (> 400 bpm). The pulse-wave Doppler signal is influenced by alignment of the ultrasound beam with the flow of blood and is heart rate dependent. Further, E/e’ is measured near the mitral annulus and consequently does not capture the global relaxation properties of the heart^21^. The acquisition of high quality B-mode images for strain analysis is largely dependent on the resolution and frame rates of the echocardiography platform, and is less dependent on the operator than Doppler methods. Post-processing of B-mode images does however incorporate some element of operator subjectivity, and development of artificial intelligence tools for strain analysis has emerged in the clinical literature to overcome this limitation^22,23^.

In the present study, T2D mice exhibited a small but statistically significant decrease in isovolumetric relaxation time. Typically prolonged (or unchanged) isovolumetric relaxation time has been reported in settings of diastolic dysfunction,^6,24^ although clinical studies suggest that severe diastolic dysfunction (Grade III) is characterized by a shortening of isovolumetric relaxation time.^25^ Mitral valve E-wave deceleration time was significantly increased in T2D mice, consistent with previous studies^5^. However, the nature of changes in both parameters are of relatively small magnitude (1–2 ms), and the underlying biological significance is difficult to assess. Deceleration time in particular has been noted for limited significance in the rodent setting of high physiological heart rates^7^. Further work needs to be undertaken to achieve consistency of measurement of these indices in rodents and to enhance the interpretation of these very small data shifts.

Early diastolic strain rate has been proposed as a measure of left ventricular relaxation, capturing the rate of ventricular wall deformation during early diastole, similar to the Doppler measure of tissue movement at the mitral annulus during the early filling phase (e’ wave). Surprisingly, e’ was unchanged in T2D mice in the present study, despite differences in diastolic strain rate. Although inter-observer variability of Doppler analysis (E/e’) was lower than diastolic strain rate (evidenced by higher Pearson correlation coefficient), the technical difficulty of acquiring tissue Doppler images in mice with small anatomy and high heart rates could be considered to be more limiting relative to acquisition of b-mode images for strain analysis. These findings suggest that, at least in this setting, diastolic strain rate is more sensitive to detection of left ventricular relaxation impairment than e’ wave measured by tissue Doppler imaging.

In the pre-clinical literature, regional analysis of myocardial wall deformation has primarily been used in studies of myocardial infarction for identifying areas of dyssynchrony in the ventricular wall^13^. In the present study, regional analysis revealed that impairment in longitudinal diastolic strain rate in diabetic mice was most prominent in the anterior free wall. Impairment in circumferential diastolic strain rate was most prominent in the inferior free wall and anterior septum. The underlying mechanism of regional diastolic impairment is not clear, but may relate to focal areas of fibrosis and/or localized thickening of the ventricular wall, and further investigation is required. These findings are similar to regional changes in myocardial deformation reported in adolescents with type 1 diabetes using cardiac MRI^26^. Significant impairment in diastolic strain rate was observed in the inferior septal and free wall regions^26^. This previous study calculated the diastolic relaxation fraction (ratio of myocardial contraction to relaxation during the filling phase^27^) and reported that adolescent T1D patients exhibit early diastolic left ventricular discoordination, despite otherwise normal cardiac function and left ventricular size. Thus assessment of regional myocardial wall deformation may provide an early indicator of diastolic dysfunction by identifying disparate regions of impairment. In the present study, regional changes in diastolic strain rate were concordant with differences detected in the global values. In future studies of more mild diastolic dysfunction, regional analysis of diastolic strain rate may be valuable to provide evidence of localized dysfunction which may not be detectable in the global output.

While left ventricular ejection fraction has been the most widely used clinical measure of cardiac function for decades, global longitudinal strain is increasingly recognized as an important clinical indicator, with prognostic value in predicting adverse outcomes in chronic systolic heart failure^28,29^. Global longitudinal strain quantifies the maximum change in length of the left ventricle during contraction (as a percentage of the initial dimension), and thus is a measure of systolic function. Interestingly, in heart failure patients where ejection fraction is preserved (HFpEF) and systolic function is generally considered to be normal, global longitudinal strain is frequently reported to be decreased^30^. In addition, there are differences in methods of measuring ejection fraction in clinical and preclinical studies. In rodents, ejection fraction is typically measured using M-mode analysis in a short-axis view, while in humans, guidelines recommend the use of biplane measurements in long-axis views^31^. In humans therefore, the longitudinal function of the ventricle will contribute substantially to the measurement of EF, while in rodents, the EF is primarily measuring short axis contraction. As long axis shortening contributes approximately 75% of stroke volume in humans, compared to a contribution of approximately 25% by short axis shortening^32^, this is likely to make EF measurements in rodents difficult to directly translate to human studies. However, in both rodents and humans, global longitudinal strain is measured in the long-axis view. In the present study, measures of systolic function acquired from the short-axis imaging plane (ejection fraction, global circumferential strain, global radial strain) were unchanged in diabetic mice. In contrast, global longitudinal strain was reduced, suggesting that longitudinal systolic dysfunction is present. These findings are consistent with other pre-clinical literature reporting that diabetic mice with normal ejection fraction exhibit reduced global longitudinal strain and diastolic dysfunction^33,34^. Clinically, global longitudinal strain has been incorporated into recently updated European Society of Cardiology guidelines for diagnosis of HFpEF^35^. In chronic settings of progressive cardiac dysfunction, such as diabetes, global longitudinal strain may provide a robust measure of early subclinical systolic dysfunction, with high predictive value, and improved translatability to clinical studies.

In the present study, both peak diastolic strain rate and peak diastolic velocity have been incorporated into the assessment of diastolic dysfunction in diabetic mice. In most cases, these variables show the same impairment, with the exception of unchanged circumferential peak diastolic velocity. By definition, strain rate is the derivative of strain, and is therefore presented as relative to the initial dimension (L_0_). Given that velocity is the rate of change of wall motion, it is therefore not dependent on the initial size of the ventricular wall. Thus, in a setting of diabetes where changes in the ventricular wall dimensions would be expected (as is the case in the present study), subtle differences in strain rate and velocity may be detectable. Additionally, there is some evidence from the clinical literature that strain rate may be more independent of acute changes in loading conditions (preload & afterload) than tissue velocities^36^, but further work is required to validate these findings in chronic settings.

### Limitations

Myocardial strain and strain rate have been reported to be influenced by blood pressure. Although blood pressure was not measured in the mice used in the present study, previous studies have reported that blood pressure is modestly elevated in mice fed a similar high fat diet^37^, thus effects of hypertension on our echocardiography values cannot be ruled out.

Our data reveal that diastolic strain rate is decreased and systolic strain rate is preserved in T2D mice. Due to variability evident in the strain data, changes in systolic measures may emerge with larger sample size.

Analysis of the passive filling phase of diastole (via Doppler or speckle tracking imaging) is notoriously difficult in small rodents with high heart rates. In the present study the early peak in mitral valve blood flow (E wave), mitral annulus tissue movement (e’) and diastolic strain rate was distinct from the atrial contraction-related peak in most cases. Some traces displayed partially fused early and late peaks, and some displayed only 1 peak in the diastolic period. Although quality images with distinct early peaks were available for every animal, replicate images where the early peak was not discernable were excluded from analysis and may have introduced an unavoidable level of bias to the dataset.

In this study echo measurements were obtained at the end of the treatment period only, and no intermediate time point data were collected. An investigation involving repeated echo measurements during the development and establishment of diabetic heart disease would certainly be informative, especially in relation to refining the mechanistic interpretation of the parameters E and e’. The potential importance of molecular studies in distinguishing the components of myocardial stiffness and impaired relaxation in driving diastolic dysfunction has been recently highlighted.^38^.

## Conclusions and clinical implications

In conclusion, here we provide evidence to support the utility of 2D speckle tracking echocardiography to document global and regional diastolic dysfunction in a pre-clinical setting of murine cardiopathology. Peak diastolic strain rate and peak diastolic velocity constitute reliable indicators of diastolic dysfunction in diabetic mice, significantly correlated with traditional Doppler measures of impaired ventricular relaxation. Some evidence of longitudinal systolic impairment was detected by global longitudinal strain, despite preserved ejection fraction. The anterior free left ventricular wall appeared to be most vulnerable to longitudinal diastolic impairment. These findings provide a significant advance on characterization of diastolic dysfunction in a pre-clinical mouse model of diabetes and offer a comprehensive suite of benchmark values for future pre-clinical cardiology studies. Given the unmet clinical need for effective treatment options for HFpEF patients, implementing diastolic strain rate analysis as a robust tool for evaluating diastolic function in mouse models will support the ongoing drug-discovery effort in this field.

## Methods

### Animals

All animal experiments were approved by the University of Auckland Animal Ethics Committee and complied with the guidelines and regulations of the Code of Practice for the Care and Use of Animals for Scientific Purposes. This study is reported in accordance with the ARRIVE guidelines (www.arriveguidelines.org). Animals were randomly assigned to experimental groups and were group housed (4 mice per cage) in a temperature-controlled environment with 12-h light/dark cycles. Food and water was provided ad libitum.

#### Type 2 diabetic mice

Type 2 diabetes was induced in C57Bl/6 J male mice by a high fat high sugar dietary intervention commencing at 7 weeks of age (n = 8 mice/group). After an initial 1 week transitional feeding period, animals were fed a high fat high sugar diet (43% kcal from fat, 200 g/kg sucrose, SF04-001, Specialty Feeds, Australia—based on the formulation of Research Diets D12451, USA) or control reference diet (16% kcal from fat, 100 g/kg sucrose, custom mouse AIN93G control diet, Specialty Feeds, Australia) for 20 weeks duration. Fresh diet was provided twice a week. This is an extensively used model of diet-induced type 2 diabetes that is characterized by obesity, glucose intolerance, mild hyperglycemia and cardiac dysfunction.^39,40^.

### Glucose tolerance testing

Glucose tolerance testing was performed after 11 weeks of dietary intervention following 6 h fasting. Baseline blood glucose levels were measured using an Accu-Check glucometer with a small blood sample from a needle prick to the tail vein. Glucose (1.5 g/kg body weight) was injected i.p. and blood glucose was measured at 5, 10, 30, 60, 90 and 120 min after the glucose injection.

### Echocardiography

Cardiac function was assessed following 20 weeks of dietary intervention. Our previous studies have confirmed that diastolic dysfunction (increased E/e’) is evident after 14 weeks of high fat high sugar diet feeding^40^, thus the 20 week timepoint allowed for sufficient time for diastolic dysfunction and other features of diabetic heart pathology to be fully established. Animals were anaesthetized with 3% isoflurane (with 1L/min O_2_ gas) and transthoracic echocardiography was performed using the VEVO LAZR-X 3100 with a MX400 probe (20–46 MHz), linear array transducer coupled with digital ultrasound system (FUJIFLIM Visual Sonics). The mice were secured to a warm platform containing ECG lead pads, and anesthesia was maintained via a nose-cone at 1.5–2.5% isoflurane (with 1L/min O_2_) throughout the procedure. All analyses were performed by an investigator blinded to study group allocation.

#### M-mode echocardiography imaging

Left ventricular (LV) M-mode two-dimensional echocardiography was performed in a parasternal short axis view at the mid papillary level to measure LV wall and chamber dimensions. LV volumes were derived from dimensions using the Teichholz equation (end diastolic volume (EDV) = (7/(2.4 + LV internal diameter at end-diastole (LVIDd))) × LVIDd; end systolic volume (ESV) = (7/(2.4 + LVID at end-systole (LVIDs))) × LVIDs). Systolic function parameters: % ejection fraction ((EDV − ESV)/EDV) × 100), and % fractional shortening ((LVIDd − LVIDs)/(LVIDd) × 100). LV mass was calculated: 1.053 × (LVIDd + LV end diastolic posterior wall thickness + end diastolic inter-ventricular septal wall thickness)^3^ − LVIDd^3^, with correction factor 0.8. At least 3 consecutive cardiac cycles were sampled per cineloop image and 3 images per animal were analyzed and averaged.

#### Pulse wave Doppler and tissue Doppler imaging

Pulse wave Doppler and tissue Doppler imaging were acquired from the apical 4 chamber view to assess LV diastolic function parameters: velocity of mitral inflow during early passive filling (E) and during atrial contraction (A), and velocity of mitral annulus during early passive filling (e’) and during atrial contraction (a’), isovolumetric relaxation time (IVRT) and E-wave deceleration time. At least 3 cardiac cycles were sampled per cineloop image and 2–4 images per animal were analyzed and averaged. Wave forms from trans-mitral and tissue records were evaluated with temporal registration to validate waveform identification.

#### Speckle tracking echocardiography

Two-dimensional echocardiography B-mode loops were acquired from the left ventricular long axis (longitudinal and radial strain) and short axis (circumferential and radial strain) views with a frame rate of 215 fps, and analyzed using Vevo strain software (VisualSonics). Endocardial and epicardial borders were traced and checked throughout three cardiac cycles to ensure optimal tracking. The time at maximum relaxation (end-diastole) was used to determine the starting phase of the strain trace. Endocardial strain and displacement analyses were performed providing global and segmental (regional) values. Endocardial strain rate and velocity parameters were derived from the strain–time and displacement–time relations respectively. The strain rate and velocity profiles for each image were examined to confirm that the early (passive filling) peak was identified by the software. In some cases, manual identification of the early peak was required, and images where the early peak was not discernable were excluded from the analysis. Average values were calculated from 2–3 independent images per animal.

### Statistics

Data are presented as mean ± SEM and statistical analysis was performed using Graphpad Prism v7.0. Statistical outliers (> 2 standard deviations from the mean) were excluded to avoid data selection bias due to stochastic measurement of sampling anomalies (likely due to digital image irregularity or motion artifact). All data sets were tested for normal distribution using Shapiro–Wilk test. Differences between two groups were analyzed by Students unpaired t-test. For analysis of body weight, glucose tolerance and regional strain parameters, a repeated measures ANOVA with LSD post hoc tests was used. For correlation analyses, Pearson’s correlation coefficient was used. A p-value of < 0.05 was considered statistically significant.

## Supplementary Information

Supplementary Information.

## Data Availability

The datasets generated and analyzed during the current study are available from the corresponding author on reasonable request.
