# Supplementary material for: Myocardial deformation imaging by 2D speckle tracking echocardiography for assessment of diastolic dysfunction in murine cardiopathology
Source: Sci Rep. 2023 Jul 31;13:12344. doi: 10.1038/s41598-023-39499-3 (PMC10390581; doi:10.1038/s41598-023-39499-3)
Supplement: Supplementary file 1 — Supplementary Information. [file 41598_2023_39499_MOESM1_ESM.pdf]

	Control	T2D
Global radial strain (%)	34.4 ± 1.7	34.1 ± 3.4
Global circumferential strain (%)	-26.9 ± 0.9	-24.6 ± 2.6
Global longitudinal strain (%)	-25.6 ± 1.2	-21.6 ± 1.2*
Longitudinal:		
Diastolic peak strain rate (1/s)	12.2 ± 0.7	8.20 ± 0.6*
Systolic peak strain rate (1/s)	-8.05 ± 0.5	-7.39 ± 0.5
Diastolic peak velocity (cm/s)	-1.09 ± 0.1	-0.74 ± 0.1*
Systolic peak velocity (cm/s)	0.84 ± 0.04	0.70 ± 0.04*
Radial:		
Diastolic peak strain rate (1/s)	-12.5 ± 0.9	-8.41 ± 0.50*
Systolic peak strain rate (1/s)	7.33 ± 0.4	7.39 ± 0.6
Diastolic peak velocity (cm/s)	-1.87 ± 0.1	-1.39 ± 0.2*
Systolic peak velocity (cm/s)	1.32 ± 0.04	1.29 ± 0.1
Circumferential:		
Diastolic peak strain rate (1/s)	12.6 ± 0.9	8.39 ± 0.8*
Systolic peak strain rate (1/s)	-8.88 ± 0.4	-9.09 ± 1.0
Diastolic peak velocity (cm/s)	-221 ± 29	-183 ± 18
Systolic peak velocity (cm/s)	206 ± 18	171 ± 20

Supplementary Table 1. Benchmark values of echocardiographic myocardial wall deformation imaging in T2D mice. Data are presented as mean ± SEM. n = 7-8/group. Analyzed by Students unpaired t-test, $*p < 0.05$.

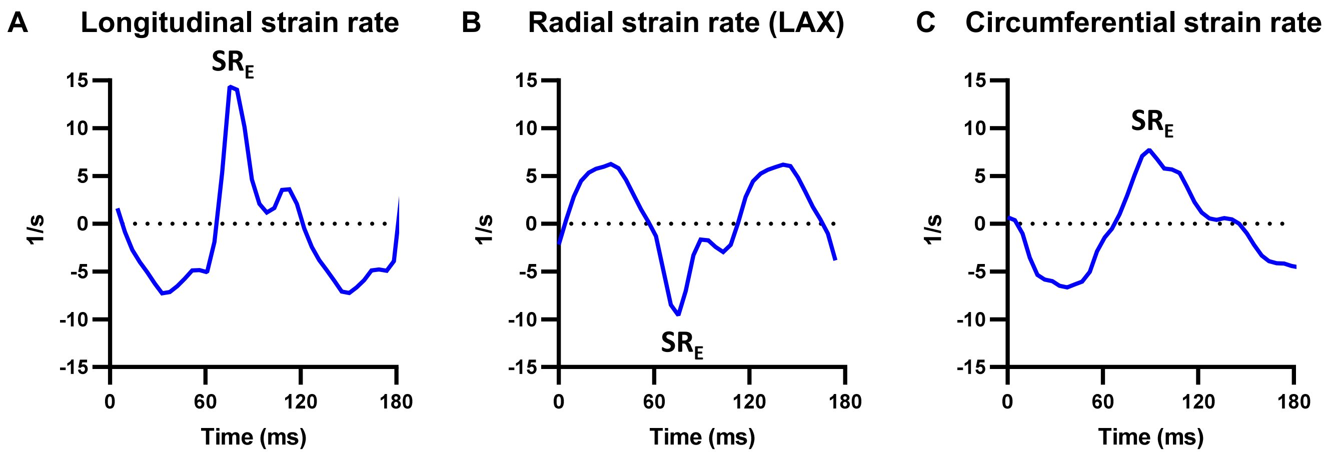

Supplementary Figure 1. Representative strain rate profiles for longitudinal, radial and circumferential planes. Early strain rate peak (E_{SR}) is indicated.

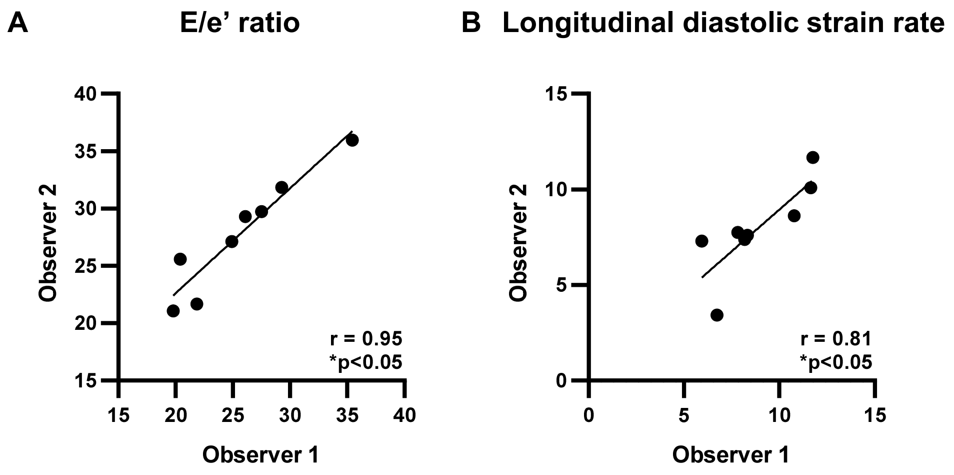

Supplementary Figure 2. Reproducibility analysis for E/e' ratio and longitudinal diastolic strain rate in mice, comparing two independent observers. r, Pearson correlation coefficient.

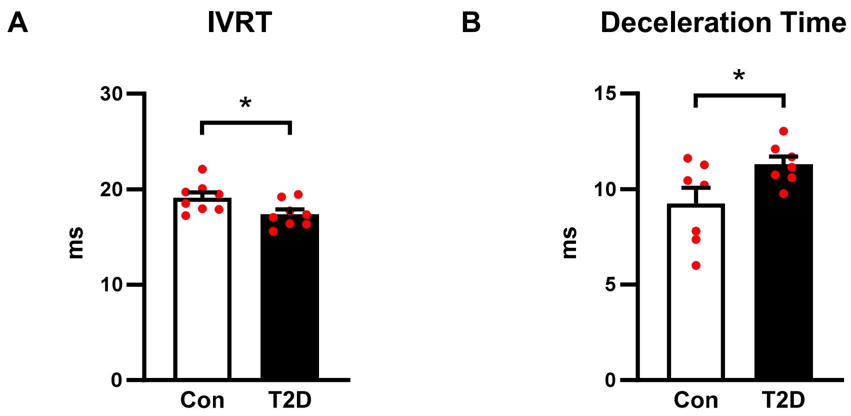

Supplementary Figure 3. (**A**) Isovolumetric relaxation time (IVRT) in T2D mice (20 weeks high fat high sugar diet). Analyzed by Students unpaired t-test. (**B**) Mitral valve E-wave deceleration time in T2D mice (20 weeks high fat high sugar diet). Analyzed by Students unpaired t-test. Data are presented as mean ± SEM. n = 7-8/group. *$p < 0.05$.
